# Impact on the Photocatalytic Dye Degradation of Morphology and Annealing-Induced Defects in Zinc Oxide Nanostructures

**DOI:** 10.1021/acsomega.2c07412

**Published:** 2023-04-17

**Authors:** Cigdem Tuc Altaf, Tuluhan Olcayto Colak, Arpad Mihai Rostas, Adriana Popa, Dana Toloman, Maria Suciu, Nurdan Demirci Sankir, Mehmet Sankir

**Affiliations:** † Department of Materials Science and Nanotechnology Engineering, TOBB University of Economics and Technology, SogutozuCaddesi No 43 Sogutozu, 06560 Ankara, Turkey; ‡ Micro and Nanotechnology Graduate Program, TOBB University of Economics and Technology, SogutozuCaddesi No 43 Sogutozu, 06560 Ankara, Turkey; § National Institute for Research and Development of Isotopic and Molecular Technologies− INCDTIM, 67-103 Donat, 400293 Cluj-Napoca, Romania

## Abstract

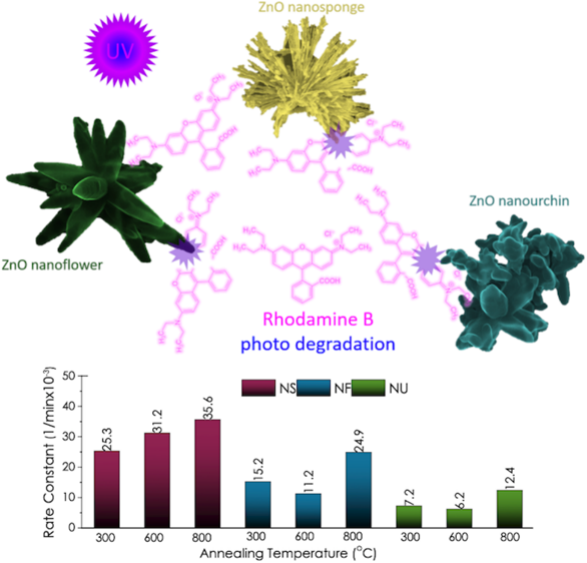

In this study, three different morphologies, nanoflower (NF), nano sponge (NS), and nano urchin (NU), of zinc oxide (ZnO) nanostructures were synthesized successfully via a mild hydrothermal method. After synthesis, the samples were annealed in the atmosphere at 300, 600, and 800 °C. Although annealing provides different degradation kinetics for different morphologies, ZnO NS performed significantly better than other morphologies for all annealing temperatures we used in the study. When the photoluminescence, electron paramagnetic resonance spectroscopy, BET surface, and X-ray diffraction analysis results are examined, it is revealed that the defect structure, pore diameter, and crystallinity cumulatively affect the photocatalytic activity of ZnO nanocatalysts. As a result, to obtain high photocatalytic activity in rhodamine B (RhB) degradation, it is necessary to develop a ZnO catalyst with fewer core defects, more oxygen vacancies, near band emission, large crystallite size, and large pore diameter. The ZnO NS-800 °C nanocatalyst studied here had a 35.6 × 10^–3^ min^–1^ rate constant and excellent stability after a 5-cycle photocatalytic degradation of RhB.

## 1 Introduction

Pristine, element-doped zinc oxide (ZnO), and the construction of heterojunctions with various semiconductors have made ground in many solar-related applications, such as energy conversion and storage, solar water splitting, solar cells, and photocatalytic water treatment.^1−9^ Especially, ZnO nanomaterials maintain their popularity due to their high optical absorption, high resistivity against photocorrosion, easy manipulation of morphology, and electrical and catalytic properties.^10−12^ Nanostructure engineering provides various morphologies with the advantages of a large surface-to-volume ratio and high electron mobility compared to bulk nanoparticles.^13−17^ Besides, the optical properties of ZnO nanostructures are highly influenced by the size, morphology, synthesis methods/reaction conditions, types of precursors, and utilization of various surfactant materials.^18−20^ Das et al. have reported that the use of trisodium citrate as a surfactant during the hydrothermal synthesis resulted in morphological changes.^19^ The presented ZnO morphologies produced in the presence of citrate exhibited novel photoluminescence properties and enhanced photocatalytic efficiency compared to ZnO formed without a surfactant. In another study, various morphologies, such as flower-like nanorods, nanoflakes, assembled hierarchical structures, and nano granules were produced via the solvothermal method in the presence of oleic acid.^21^ Consequently, the type and amount of surfactant used in synthesis have been proven to be very influential on morphology, size, and optical properties such as band gap energies and photoluminescence.^22^

Another issue that has come to the fore, especially in recent years, is that ZnO materials’ optical, electrical, and catalytic performances vary according to their defect structures.^23−28^ It has been known that excess zinc and oxygen vacancies form donor states at 0.05 eV below the conduction band.^29,30^ Oxygen vacancies (O_v_), Zn interstitials (Zn_i_), and hydrogen background impurities have been suggested as candidates for native donors in ZnO.^30^ There has been a great discussion about the production conditions of native donors in ZnO and their electrical and optical properties.^31−33^ Halliburton et al. have reported that annealing ZnO crystals at 1100 °C in zinc vapor increased n-type electrical conductivity.^31^ They claimed that the neutral oxygen vacancies are responsible for the absorption band in the blue region that causes the red appearance after annealing, and either zinc interstitials or neutral oxygen vacancies are responsible for increasing free carriers. Bandaru et al. have studied the annealing-induced transformation and/or enhancement in the electronic defect states for the aluminum-doped ZnO thin films deposited on a soda lime glass substrate.^32^ They concluded that annealing under atmospheric pressure resulted in deep donor-level defects in the form of only excess oxygen. On the other hand, annealing at reduced pressure created single-charged oxygen vacancies and shallow donor-level defects. Various annealing temperatures (150–900 °C) and atmospheres, including hydrogen, argon, and nitrogen, have been reported to deploy the defect-related properties of ZnO.^33−39^ For instance, annealing of ZnO under an N_2_ atmosphere at 550 °C bears more Zn_i_ and fewer O_v_, which results in stronger blue and green emissions.^39^

The photocatalytic performance of ZnO catalysis has also been affected by the nature of native defects. For instance, enhanced surface defects for ZnO nanostructures produced by annealing at 500 °C have been reported to reduce recombination of the photogenerated charge carriers and, thus, enhance efficiency for photocatalytic rhodamine B degradation.^38^ Although it is known that better photocatalytic performance is obtained by increasing the charge carrier concentration and light absorption capacity by defect engineering in ZnO nanostructures, there is no established understanding of the control of defect structures with experimental conditions in different morphologies. Therefore, studies are still ongoing on postproduction processes and defect engineering. Bose et al. used the electrochemical technique to deposit hexagonal and tapered ZnO nanorods on the glass substrate.^40^ It has been understood that this small morphological difference plays an important role in photocatalytic performance by triggering the formation of lower energy band levels by defect formation in the ZnO structure and thus capturing photogenerated electrons and holes.^40^ O_v_ and Zn_i_ play a significant role in the photocatalytic activity of ZnO.^41,42^ Recently, P. Nandi and D. Das reported that the defect states in ZnO lower the fast recombination of electrons and holes, increase charge transport and accelerate the photocatalytic activity.^43^ They reported that it is possible to incorporate oxygen into ZnO structures calcined at temperatures of 600 °C and above, affecting the photocatalytic activity. In other words, it has been reported that increasing the number of surface defects and the reactive surface area improves electron–hole segregation efficiency. Thus, superior photocatalytic activity is obtained in ZnO nanostructures.

Considering previous studies reported in the literature, this study aims to describe the influence of structural morphology and defect properties on photocatalytic activity. The effect of morphological variations obtained from hydrothermal synthesis of ZnO nanostructures having three different morphologies (nanoflower (NF), nano urchin (NU), and nano sponge (NS)) with postannealing at 300, 600, and 800 °C under atmospheric conditions was investigated by electron paramagnetic resonance (EPR) spectroscopy to build a relationship with their photocatalytic activity. Photoluminescence, energy dispersive X-ray spectroscopy, transmission electron microscopy, Fourier-transform infrared spectroscopy, and UV–visible absorption spectroscopy were used to support our findings. In addition to the morphology control, which can be easily performed with the hydrothermal method, we proved with this study that effective catalysts for the photocatalytic degradation of rhodamine B can be developed by defect engineering via annealing in atmospheric conditions. We see that the catalysts and production methods we have developed are suitable for mass production and contribute to the literature due to their performance and cost-effectiveness.

## 2 Experimental Section

### 2.1 Materials

Zinc nitrate hexahydrate (Zn(NO_3_)_2_·6H_2_O, Sigma-Aldrich), Poly(ethylenimine) (PEI) solution (Sigma-Aldrich, MW ∼750,000 in H_2_O), nitric acid (HNO_3_, Sigma-Aldrich), ammonium hydroxide solution (NH_4_OH, Sigma-Aldrich), sodium sulfide (Na_2_S, Sigma-Aldrich), an urea (CH_4_N_2_O, Sigma-Aldrich) were purchased and used in the experiments.

### 2.2 Synthesis of the Nanoparticles

Hydrothermal growth of ZnO nanoparticles having various morphologies has been performed by altering the precursors and reaction conditions described in our previous works.^17,20,44,45^ Zn(NO_3_)_2_·6H_2_O has been used as a zinc source, while NH_4_OH and urea have been used as precursors for ZnO nanoflower (NF) and nano sponge (NS) samples, respectively. Urchin-like ZnO (NU) has been synthesized by adopting the NF synthesis in the presence of a polyethylenimine (PEI) solution. Briefly, NF powder has been synthesized using 0.1 M Zn(NO_3_)_2_·6H_2_O and 0.15 M NH_4_OH at 80 °C for 1 h. On the other hand, NS powder has been prepared from a 100 mL aqueous solution of 0.05 M Zn(NO_3_)_2_·6H_2_O and 1.0 M urea. The pH value of this reaction solution has been set to 5.4 with concentrated nitric acid. Then, the reaction solution was kept in a standard reaction oven at 80 °C for 3 h.

NU has been synthesized in two steps of hydrothermal growth (described above) using the same amounts of the precursors of NF but with the addition of a PEI solution (0.75 μg·mL^–1^). The product from the standard hydrothermal reaction of NF has been collected with filtration, rinsed with distilled water/ethanol, and dried. The powder was treated with a 1 M Na_2_S solution for 2 h by stirring, filtering, and washing with distilled water, followed by calcination at 500 °C for 3 h. The calcinated powder was added to the fresh hydrothermal solution containing 0.1 M Zn(NO_3_)_2_·6H_2_O, 0.15 M NH_4_OH, and PEI and kept at 80 °C for 1 h. After the reaction, the product was filtered and rinsed with distilled water/ethanol. Each morphology was calcinated at 300, 600, and 800 °C for 30 min and labeled according to morphology and calcination temperatures.

### 2.3 Characterizations

X-ray diffraction (XRD) analysis has been achieved for powder samples using PANalytical/Philips X’Pert MRD system. Fourier-transform infrared (FTIR) analysis has been conducted using a PerkinElmer Spectrometer 100. Investigations of the contents of the defect centers and the light emission performances of the nanoparticles have been analyzed via a Horiba Jobin-Yvon Florog-550 photoluminescence (PL) system under a 325 nm He:Cd laser excitation wavelength. BET analysis has been performed to collect pore size and surface area measurements using Nova Quantchrome 2200e. Electron paramagnetic resonance (EPR) spectroscopy measurements were carried out on a dual-band E500 ELEXSYS (Bruker) spectrometer operating at a microwave frequency of 9.877 GHz, combined with an M365FP1 UV-diode (Thorlabs), which was used for irradiation (λ = 365 nm with a power of 9.8 mW). The generation of reactive oxygen species by ZnO under UV irradiation was tested using EPR and the well-known spin-trapping method. 5,5-Dimethyl-1-pyrroline *N*-oxide (DMPO) was used as a spin-trapping agent. Scanning transmission electron microscopy (STEM) and transmission electron microscopy (TEM) measurements were carried out on a Hitachi HD 2700 microscope at a 200 kV electron acceleration. The samples were deposited on a 300-mesh copper electrolytic grid, to which a carbon film was attached. The sample was cleaned before being introduced into the electron microscope with a TEM ZONE, which removes unattached parts of the sample from the grid. The photocatalytic activity of the samples was evaluated in a homemade laboratory reactor system using a UV lamp (30 W) which emits at λ = 365 nm, or a 400 W halogen lamp (Osram). A synthetic solution of rhodamine B (RhB) (10 μM) was used as a pollutant. The catalyst was suspended in an aqueous solution of RhB. The mixture was stirred in the dark for 1 h to achieve the adsorption–desorption equilibrium. Each experiment was conducted continuously for 3 h, and at every 60 min, 3.5 mL of the solution was withdrawn for analysis. The catalyst was separated from the suspension by centrifugation. The resulting solution was analyzed with a UV–vis spectrophotometer by recording the specific absorption maximum of the pollutant (at 553 nm). Photocatalytic activity is calculated using the following equation:
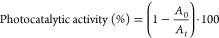
where A_t_ and A_0_ represent the RhB absorbance at 553 nm at time *t* and at *t* = 0, respectively. The apparent rate constant, *k*_i_, was calculated using the first-order kinetic model to describe the RhB photocatalytic degradation process, which is given by
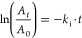


## 3 Results and Discussion

**Figure 1:**
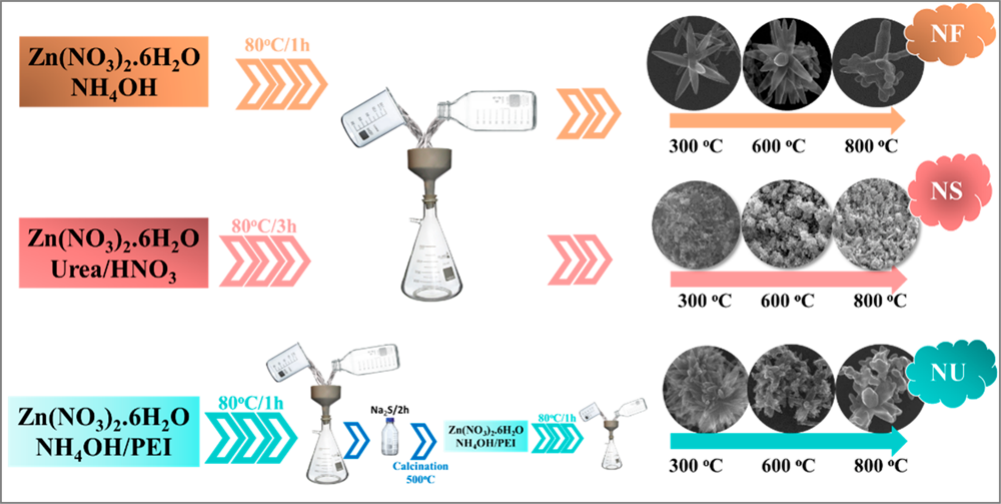
Summary of the experimental procedure, including SEM images of various ZnO nanostructures.

The present study investigated the influence of three different hydrothermal reaction solutions and annealing temperatures on ZnO’s morphology, growth, and defect structure. The powder sample collected from the reaction solution of a hydrothermal reaction containing NH_4_OH and Zn(NO_3_)·6H_2_O at 80 °C for 1 h resulted in a flower-like ZnO (NF). On the other hand, an urchin-like structure (NU) has been obtained from the treatment of the NF powder in Na_2_S, followed by calcination and second hydrothermal growth under the same conditions. Preparation in a slightly acidic solution (5.4 pH) containing Zn(NO_3_)·6H_2_O, urea, and nitric acid at 80 °C for 3 h resulted in a sponge-like morphology (NS) of ZnO. All these nanostructured ZnO powders have been annealed at 300, 600, and 800 °C for 30 min under atmospheric conditions to enhance the materials’ characteristics such as crystallinity, defect structures, and optoelectronic properties. Figure 1 summarizes the experimental procedure.

**Figure 2:**
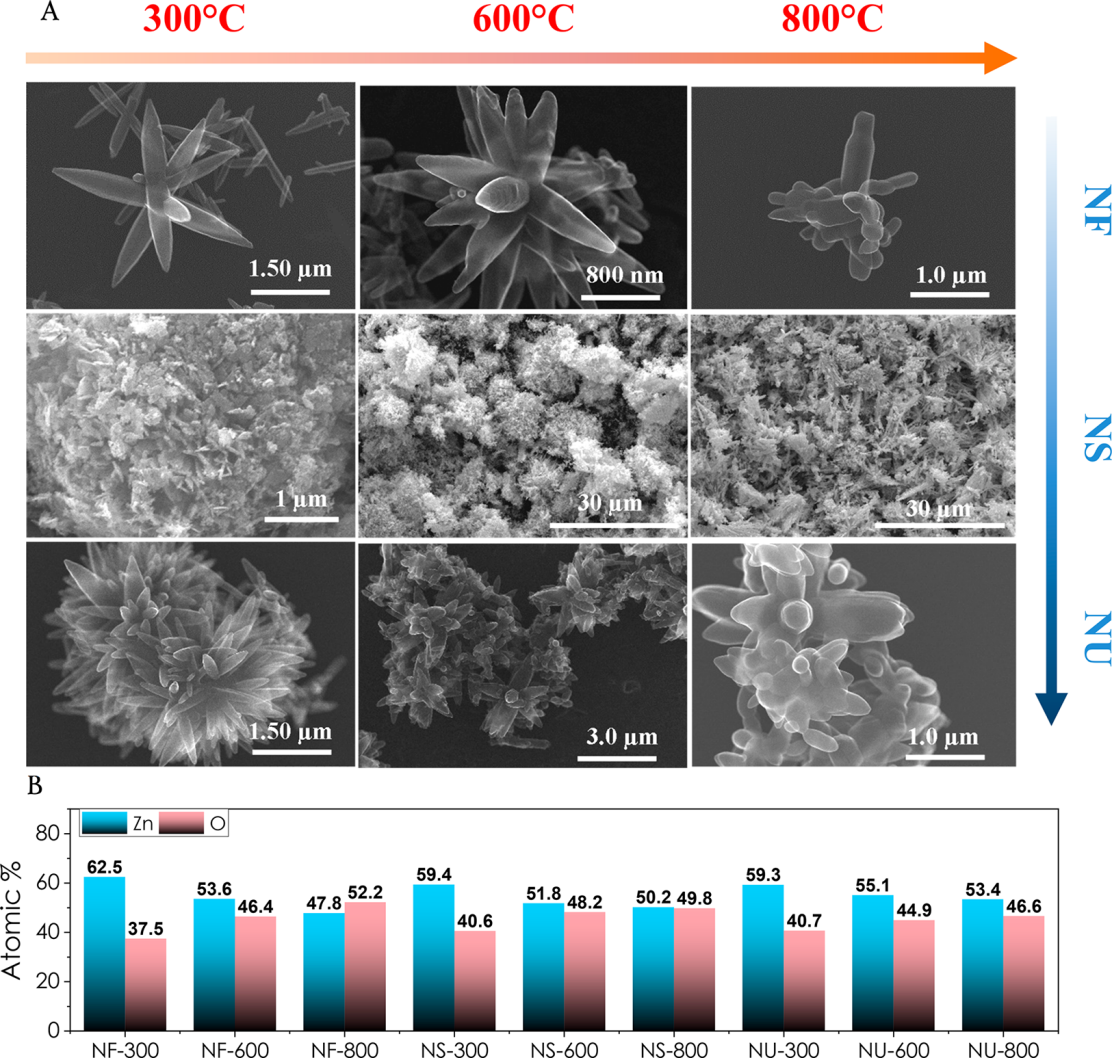
(A) SEM images and (B) atomic percentage values of the ZnO powder samples.

Figure 2A shows scanning electron microscopy (SEM) images and atomic percentage values of each element calculated from energy dispersive X-ray (EDS) analysis in the ZnO powder samples having different morphologies after annealing. As depicted in the SEM images, the ZnO NF structure forms a bunch of well-shaped nanowires combining to form a flower-like shape. The well-shaped nanowires mostly preserve as the temperature increases to 600 °C. On the other hand, the shape of the nanostructure becomes worm-like after annealing at 800 °C. The synthesis process in a slightly acidic medium (pH 5.4) results in small nanoparticles of ZnO forming sponge-like morphology, and it is observed that the morphology remains unchanged during annealing. On the other hand, the urchin-like morphology of ZnO (NU) forms through a modified reaction of ZnO NF using PEI as stabilizing/capping agent.^46^ The same as NF, this morphology forms as bunches of nanowires but smaller and thinner due to the presence of PEI. It has been reported that the addition of PEI influences the density of nanowires owing to the alteration in the nucleation process of the ZnO seed layer.^47^ During the preparation of NU, additional chemical treatment with Na_2_S has been applied to obtain close-packed ZnO formations. In general, Na_2_S treatment has been reported to form ZnO–ZnS heterostructures in the literature.^48,49^ Inserting ZnO powder or thin film in Na_2_S solution results in an ion-exchange reaction and forms a ZnS layer on ZnO. In this study, the ZnS layer has been converted to another ZnO layer by annealing at 500 °C. This new ZnO layer supplied medium to form new close-grained ZnO NU morphology. The increase in annealing temperature results in slight deformation of the ZnO wires. Atomic percentage values of the Zn and O elements obtained from EDS analysis (Figure 2B**)** depict a clear increase in oxygen amount with increasing temperature.

**Figure 3:**
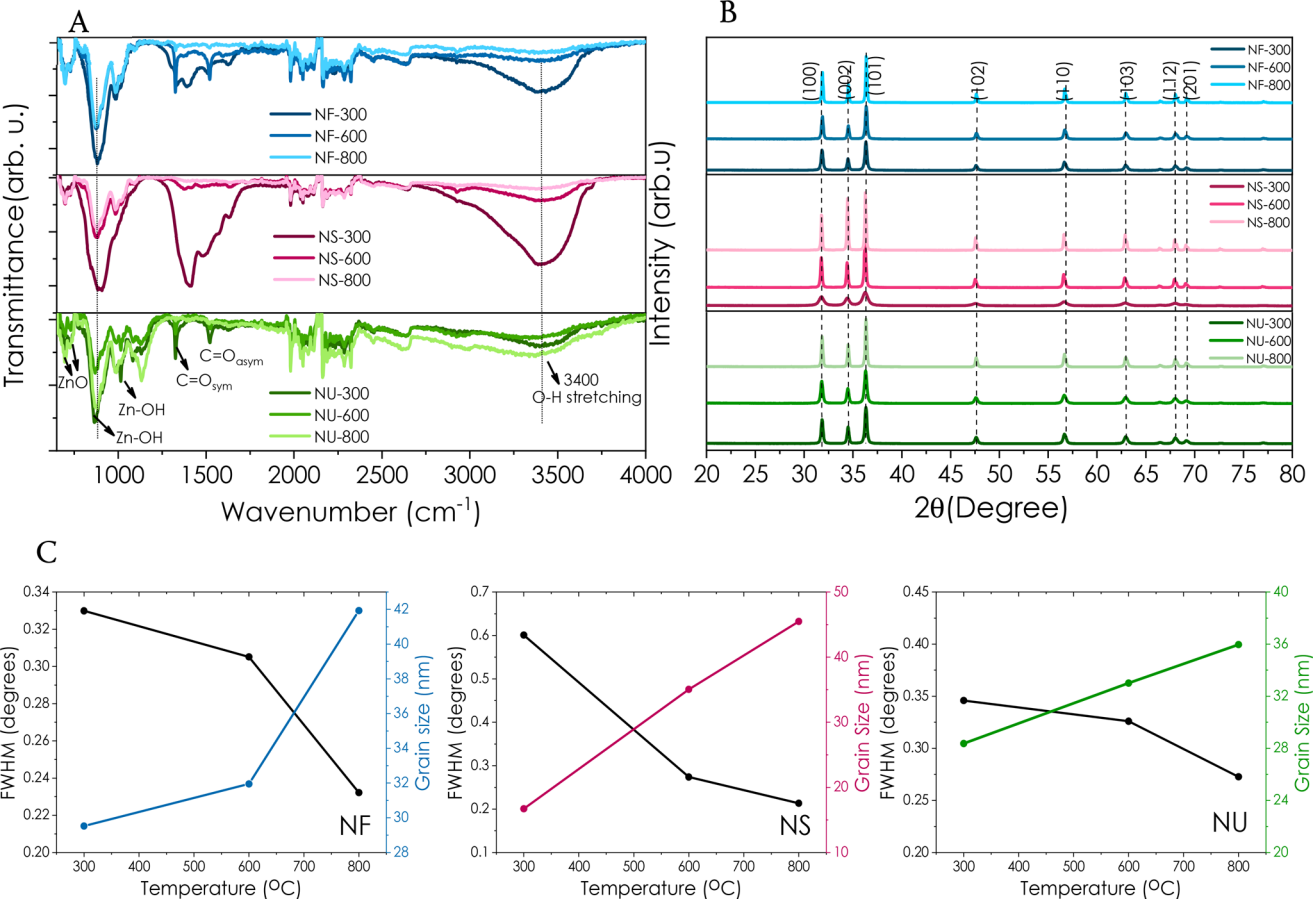
(A) FTIR, (B) XRD spectra, (C) average crystallite size and full width at half maximum (FWHM) values depending on morphology and annealing temperature.

The FTIR spectra of ZnO nanostructures are depicted in Figure 3A. All the morphologies exhibit a broad peak at ∼3400 cm^–1^ corresponding to the O–H stretching, showing the presence of hydroxide groups in all the samples.^50^ The peaks at 1320 and 1514 cm^–1^ correspond to C=O symmetric and asymmetric stretching of carboxylate ions, respectively. The intensities of these two peaks decrease with increasing annealing temperature, indicating the complete transformation of Zn_5_(CO_3_)_2_(OH)_6_, which is the crude product of the hydrothermal reaction of urea and zinc nitrate to ZnO.^17^ Finally, the stretching mode of the Zn–O bond is observed in the range between 650 and 740 cm^–1^.^51,52^ Changes in the crystal structure and phase purity of the synthesized ZnO nanoparticles with morphology and annealing temperature have been determined via XRD measurements as given in Figure 3B. X-ray powder diffraction verified a single-phased ZnO formation for each sample. All diffraction peaks are well indexed with the hexagonal phase of ZnO wurtzite crystal structure with (100), (002), (101), (102), (110), (103), (112), and small (201) and (202) crystal planes (JCPDS 36–1451).^20,45^ On the other hand, the intensities of the peaks vary with the annealing temperature for all morphologies,^53^ which is most probably owing to the improvement in crystallinity of the samples as smaller grains inosculate to form larger ones, leading to an increase in average crystallite size as the temperature increases.^37,54^ The crystallite size has been estimated using Scherrer’s equation for all samples, and the largest crystallite size has been observed at 800 °C with 50.97, 50.94, and 44.40 nm for NF, NS, and NU, respectively (Figure 3C). Williamson–Hall analysis results for ZnO having different morphologies and annealing temperatures have been given in Figure S1. Table S1 displays the comparative table for crystallite size and lattice strain changing with morphologies and annealing temperature.

N_2_ sorption results show the samples’ type II and IV isotherm behavior (Figure S2) and porous structures.^55−57^ Generally, this hysteria type is observed for nanoporous powders. Average pore sizes, calculated using BJH Theory, are observed between ∼2.1 and 1.4 nm (Figure S3, Table S1). The results show that the average particle size of the ZnO powders decreases with annealing temperature, whereas pore diameters increase. The increase in pore diameter at higher temperatures is attributed to the formation of larger grains, supporting the XRD analysis results. The ZnO having NF morphology exhibits type II adsorption with an H4-type hysteresis.^55,57,58^ As smaller pores collapse during the melting of ZnO, structures turn into bulkier crystals, and the empty space between them starts to dominate. NS and NU, on the other hand, show different trends under the same conditions. NS shows a type IV isotherm with an H3-type hysteria^56,59^ at higher relative pressures. There is a significant grain size increase, surface area decrease, and pore radius increase with increasing annealing temperature.

**Figure 4:**
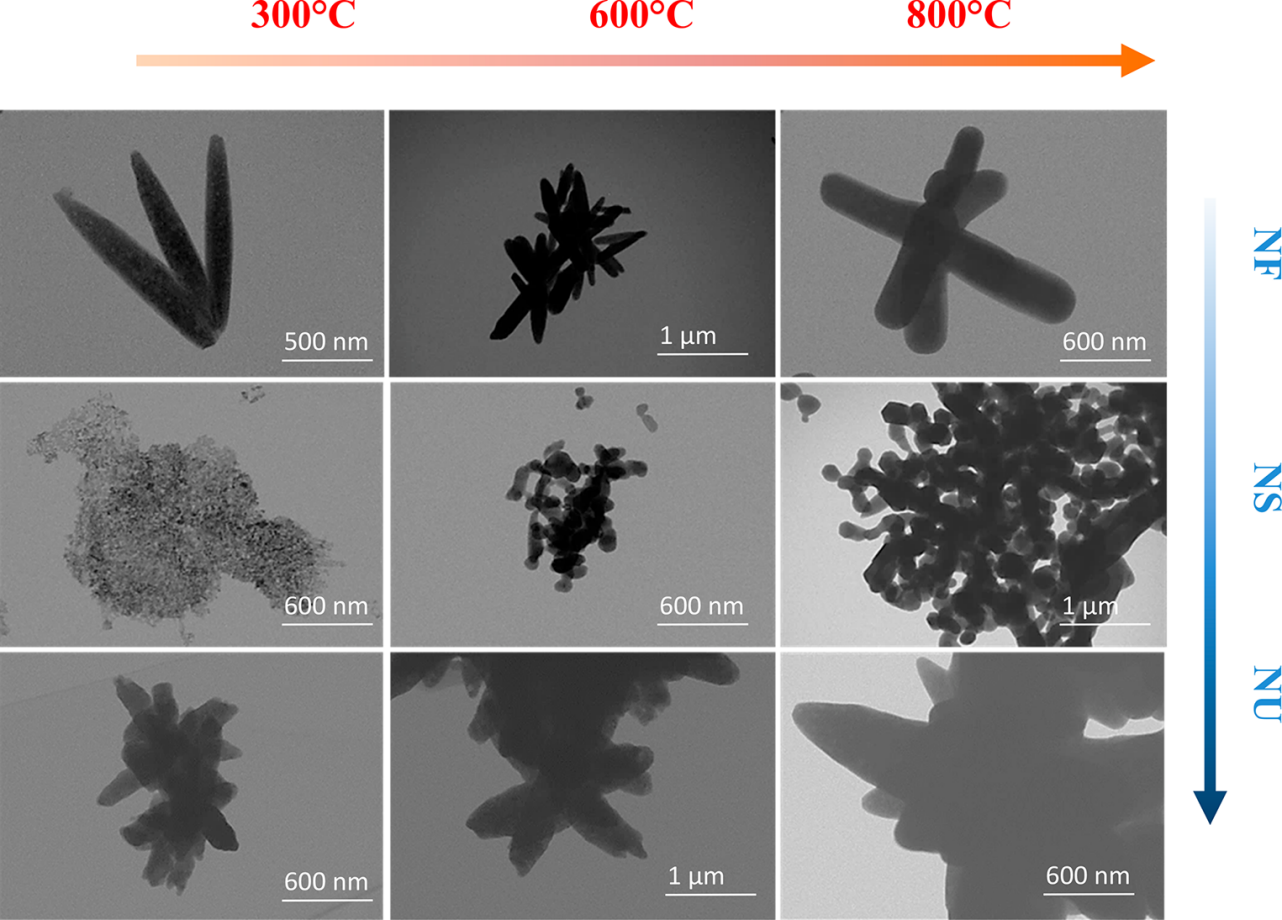
TEM images of the ZnO powder samples.

BET analysis and BJH pore size distributions imply that NS-300 has mesopores along with nanopores. NS particles form their sponge-like structures, and as they melt, they start forming smaller spheres that gather around each other and form larger sphere-like structures. Despite having a low surface area, nanosponges have two significant pores structures of 2 and 5.5 nm pores in diameter, at 800 °C; also, at this temperature, ZnO starts to form sponge-like spheres. NUs are much like NFs, except they have smaller pore diameters at higher temperatures. They also show a type II isotherm behavior with an H4-type hysteresis and nanoporous pore structure.^58,60,61^ They resemble nanoflowers in morphology at lower temperatures, only in a denser form. At higher temperatures, they decrease like other samples; however, the size of their average pores remains lower. TEM images of the ZnO nanoparticles are displayed in Figure 4. The NF structure is confirmed to be formed of wire agglomerations of approximately 700 nm in length and with thin tips. Upon annealing at 800 °C, the thin tips become round. TEM image of NS samples confirms the formation of tiny nanoparticles agglomerate to form a sponge-like structure.

### 3.1 Electron Paramagnetic Resonance Spectroscopy Results

**Figure 5:**
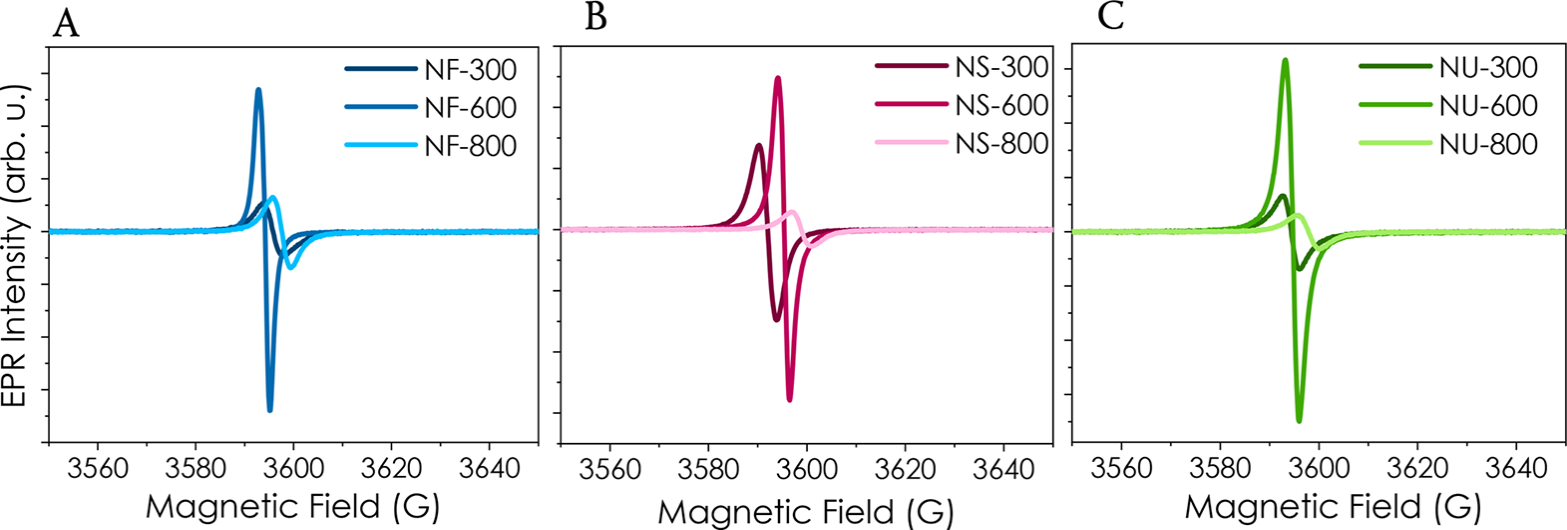
EPR spectra of the ZnO samples with different morphologies (A) NF, (B) NS, and (C) NU annealed at 300, 600, and 800 °C.

EPR spectroscopy measurements results, carried out on the ZnO samples with different morphologies (NF, NS, and NU) annealed at different temperatures (300, 600, and 800 °C), are presented in Figures 5A, B, and C, respectively. All spectra show a good, resolved signal with varying g-values around g ∼ 1.96 (see Table 1), which are characteristic of ZnO core defects.^62^ At the same time, no surface defects, which normally have a g-value around g = 2,^62^ were detected, indicating that the materials have a defect-free surface and that the core defects are dominant in the studied ZnO nanomaterials. Since the same amount of sample was used for the EPR measurements, a direct comparison between the signal intensities is possible, showing that the samples annealed at 600 °C present the highest concentration of core-defect centers, indicating a disordered structure of the materials. By increasing the annealing temperature to 800 °C, the core defect concentration drops significantly, demonstrating better homogeneity and defect-free samples. As a general trend, UV irradiation did not significantly affect the EPR intensity (Figure S4).

**Table 1:** G-Values of the ZnO Samples with Different Morphologies (NF, NS, and NU) Annealed at 300, 600, and 800 °C, Respectively

	g-values		
Sample	300 °C	600 °C	800 °C
NF	1.96228	1.9631	1.96123
NS	1.96407	1.96232	1.96031
NU	1.96211	1.96309	1.96129

### 3.2 Optical Properties

**Figure 6:**
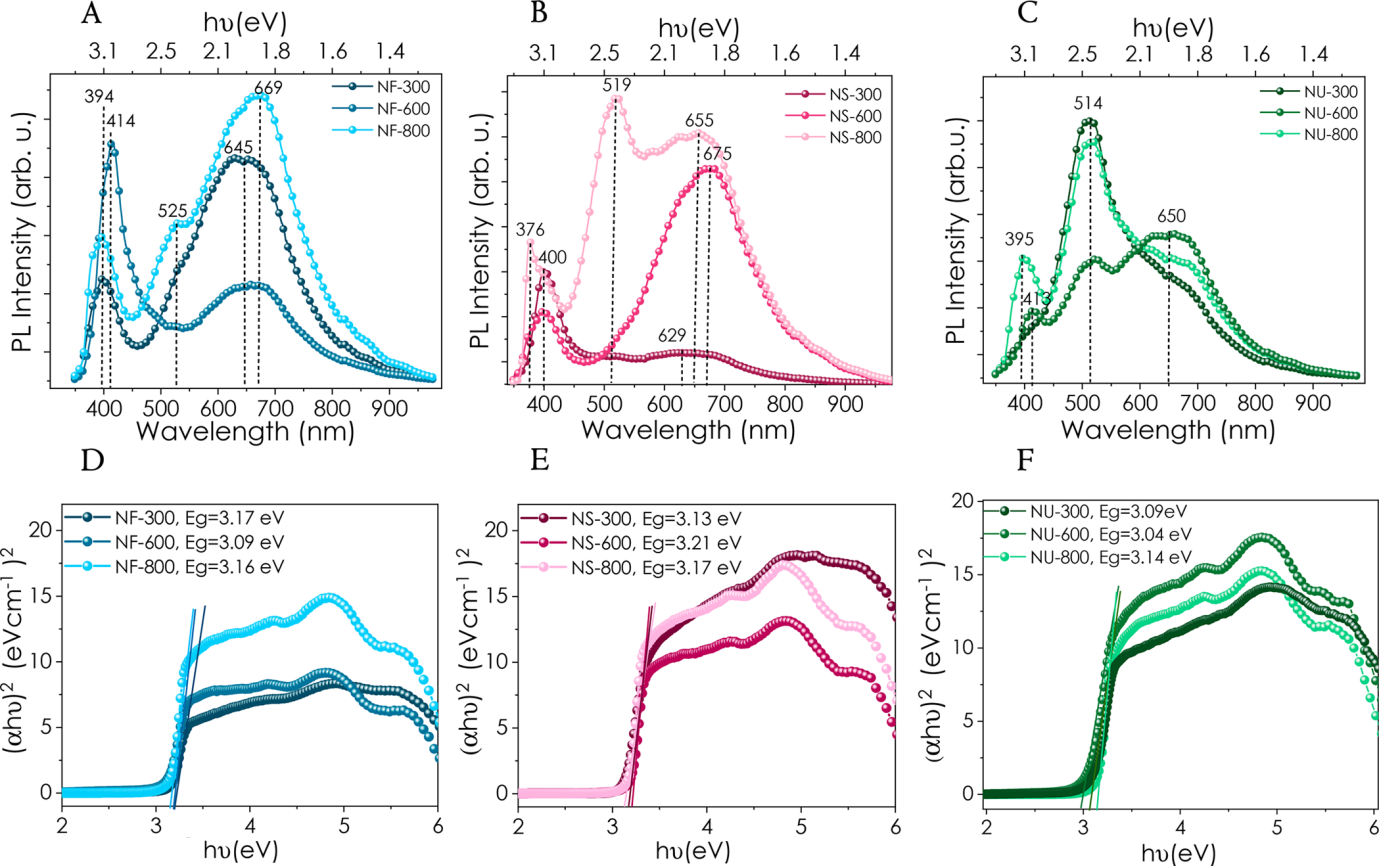
(A–C) Room temperature PL spectra and (D–F) Tauc plots of ZnO powders depending on morphology and annealing temperature.

Room temperature PL study is one of the most viable techniques to confirm the nature of the defects of ZnO nanoparticles. Most commonly, the PL spectrum of ZnO consists of two emission bands: (i) at around 372–400 nm in the UV region, which corresponds to the near-band-edge emission (NBE) excitonic UV emission, and (ii) a broad deep-level emission (DLE) residing at around 450–800 nm.^52,54,63^ The center of the peak related to NBE emission shifts to higher wavelengths, indicating the narrowing band gap values (∼3.0 eV) for NF and NU morphologies, as the annealing temperature increases from 300 to 600 °C, consistent with the Tauc graphs. The donor impurities can explain the decrease in the band gap values, as they generate energy levels near the conduction band edge.^54^ The findings of the PL studies corroborate the EPR results (Figure 6A–C), as the maximum defect signals are observed for the samples annealed at 600 °C. However, the PL character of the NF and NU morphologies return to their initial stages as the annealing temperature rises to 800 °C, where similar PL spectra are observed for the samples annealed at 300 and 800 °C. On the other hand, the NS samples behave inversely compared to the other two, and the band gap increases slightly with increasing annealing temperature. Besides NBE emission in the UV region, visible emission is crucial for ZnO. It is related to the defect levels (DLE), including oxygen and zinc defects, zinc vacancies, and interstitial zinc. DLE peaks cover an extended region (450–850 nm) for all morphologies. The widening DLE signals of ZnO morphologies are due to the grain growth with increasing annealing temperature. As reported in earlier studies, annealing temperatures and morphology significantly influence the type and concentration of defects in the ZnO nanostructures.^25,64,65^ At high-temperature annealing (800 °C), all morphologies show the existence of neutral oxygen vacancies (V_O_) located at ∼650 nm. It is also observed that the DLE based on doubly charged oxygen vacancies (V_O_^++^) emission reaches the highest point as the temperature increases. This type of defect is minimal for NS morphology annealed at 300 °C. An energy level diagram showing observed defect levels in ZnO nanoparticles at 300 °C has been schematically displayed in Figure S5. The Tauc relation was used to evaluate the ZnO nanoparticles’ optical band gap energy (E_g_) with different morphologies and annealing temperatures.^52^ The UV–vis absorbance spectra of NS-300, NF-300, and NU-300 samples can be seen in Figure S6. Compared to the band gap of the bulk ZnO (∼3.3 eV),^66^ all the morphologies exhibited smaller band gap values (Figure 6D–F). Although all morphologies have similar band gap values, the NU structure has a slightly smaller band gap value, possibly due to the incorporation of PEI during the synthesis.

### 3.3 Photocatalytic RhB Degradation

**Figure 7:**
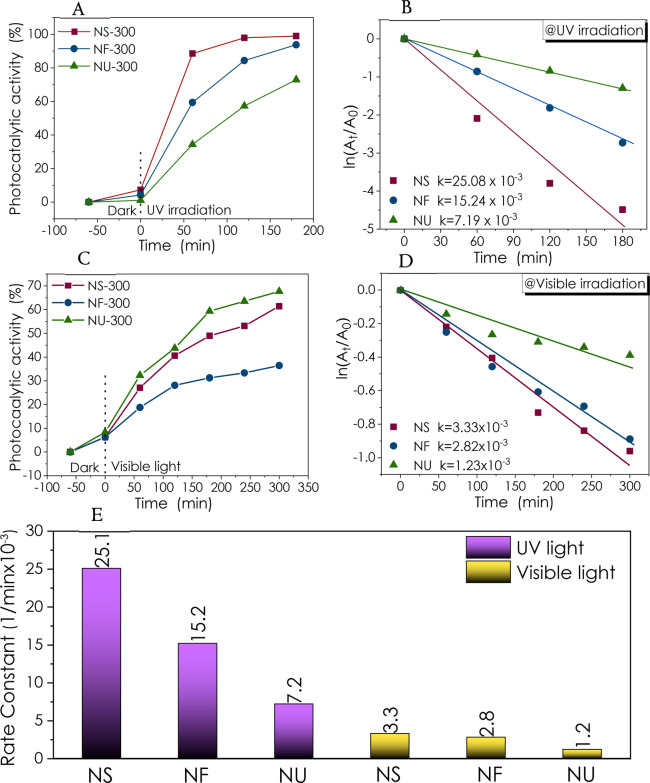
Comparison of RhB degradation at (A–B) UV and (C–D) visible irradiation at 300 °C. (E) Comparison of rate constant values for ZnO morphologies at 300 °C.

It is a well-known fact that the photocatalytic activity of ZnO depends on its surface properties.^17,67,68^ Therefore, ZnO nanopowders with different morphology and defect properties were used in the photocatalytic degradation of the RhB organic dye, a water-soluble fluorescent xanthene dye used to dye various materials. Due to its toxicity, much effort has been paid to remove or depredate RhB into harmless species.^69−72^ Five mg of ZnO nanopowder was dispersed in 10 mL of dye solution. The changes in the optical absorption spectra in time were recorded (Figure S7) to understand the effect of the morphology and defects on the photocatalytic degradation of RhB; Figures 7A–E compare the RhB degradation performance of ZnO NS, NF, and NU annealed at 300 °C under atmospheric conditions. ZnO NS nanopowders depict superior performance compared to other morphologies. The rate constants of 25.08 × 10^–3^, 15.24 × 10^–3^, and 7.19 × 10^–3^ min^–1^ have been calculated under UV illumination for ZnO NS, NF, and NU, respectively. Photocatalytic RhB degradation performance for all samples has been summarized in Table S2.

On the other hand, the photocatalytic activity of the ZnO NS was better than that of other morphologies under visible light (Figure 7C). In terms of kinetic work, as expected, UV illumination resulted in higher rate constants for all samples compared to visible light illumination. Heterojunctions can be built to extend the optical response of the ZnO nanopowders through the visible region.^73,74^

**Figure 8:**
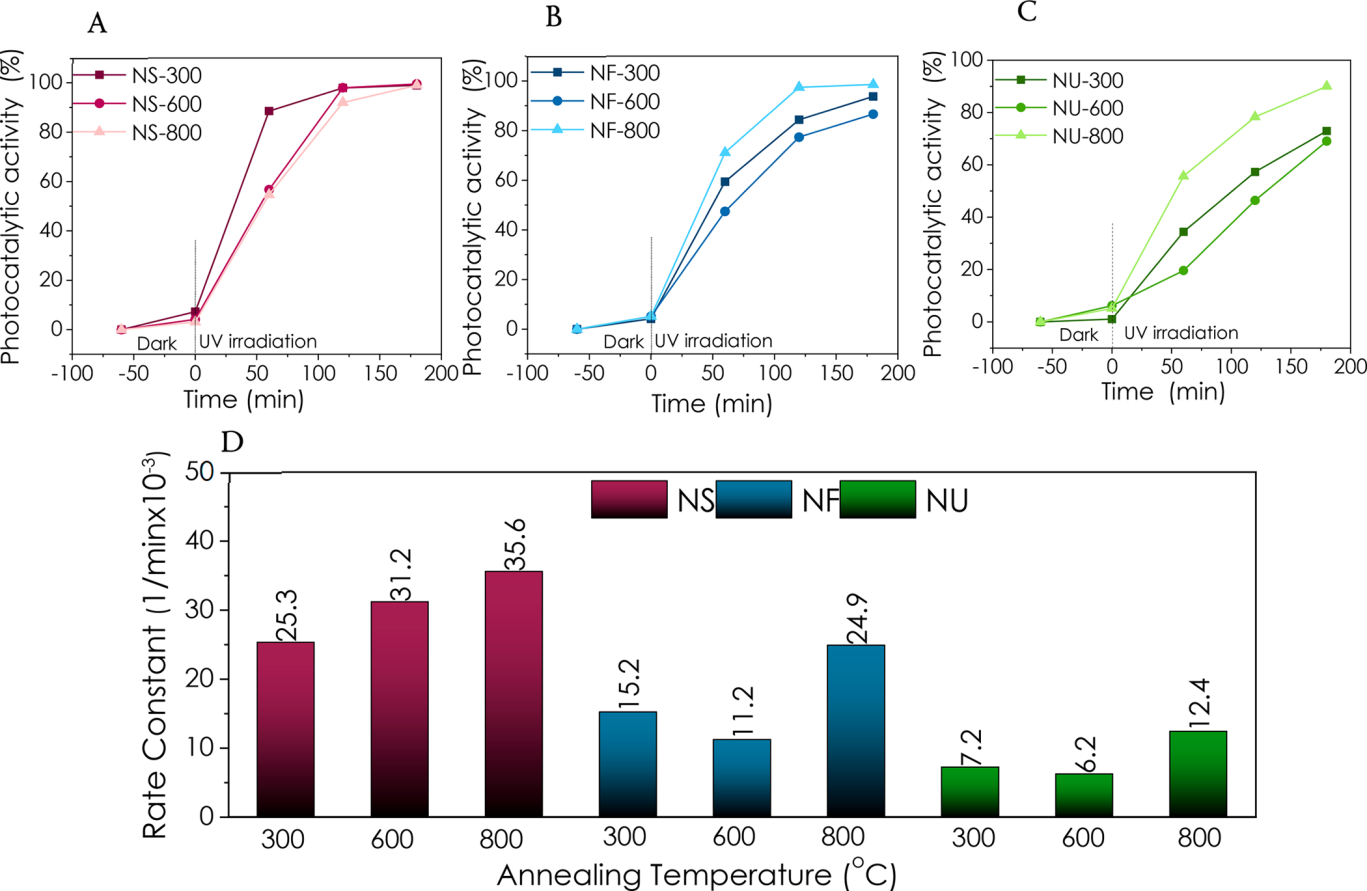
Photocatalytic RhB degradation at UV irradiation for (A) NF, (B) NS, and (C) NU annealed at 300, 600, and 800 °C. (D) Comparison of rate constant values of the morphologies depending on the annealing temperature.

As shown in Figure 8, the ZnO samples’ morphologies and annealing temperature influence the degradation performances differently. Although annealing provides different degradation kinetics for different morphologies, ZnO NS performed significantly better than other morphologies for all annealing temperatures used in this study. The 35.6 × 10^–3^ min^–1^ rate constant obtained for the ZnO NS annealed at 800 °C is one of the highest values for bare ZnO catalysts reported in the literature (Table 2). When the photocatalytic activity after 120 min is compared, the performance of the ZnO catalysts with different morphologies under UV illumination shows that NS, NF, and NU have activities of 98, 97.7, and 79%, respectively. Therefore, it can be concluded that the degradation performance of NS and NF is very close, but ZnO NU does not perform as well. For the best-performing catalyst in this work, ZnO NS-800 °C, with a grain size of 45.5 nm and pore size of 20.73 Å, are among the pronounced differences compared to the other samples (Table S1). Besides, as evidenced by EPR, all samples’ signals from the core defects have been lowered via annealing.

**Table 2:** Comparison of Rate Constants of Bare ZnO Catalysts for Photocatalytic Degradation of RhB

Reference	Rate Constant (min^–1^)
This work	0.0356
J. Wang et al.^79^	0.0066
Q. I. Rahman et al.^80^	0.0343
M.A. Alvi et al.^81^	0.0246
P. Nandi et al.^43^	0.042
S. Kumar et al.^82^	0.0039

On the other hand, PL intensities for NBE and DLE are enhanced by air annealing. It is known that the photocatalyst’s light absorption and photocatalytic properties strongly depend on several factors, such as surface area, morphology, particle size, and crystallinity.^75,76^ Studies on various ZnO morphologies have proved that the abundance of specific sites strongly depends on the morphological variations and preparation route.^76^ Extended literature research shows that these factors sometimes may compete with each other to influence photocatalytic activity, and thus, there may be some conflicts within the findings. In recent studies, the relationship between photocatalytic performance and defect structures in ZnO has been explored with EPR technology.^77^ For instance, in photocatalytic RhB degradation using ZnO, it has been reported that oxygen and/or zinc vacancies negatively impact the photocatalytic properties, and fewer surface defects result in higher photoactivity.^77^ In another study, it was observed that the interstitial zinc defects were influential in promoting the photogenerated electron–hole pairs separation and, thus, increased ZnO photocatalytic activity.^78^ In light of all these results, it can be concluded that the superior performance of ZnO NS-800 °C can be attributed to multiple factors, including a lower concentration of core defects, higher NBE and DLE densities, better crystallinity, and larger pore size compared to the NF and NU samples.

### 3.4 Photodegradation Mechanism

#### 3.4.1 Scavenger Tests

Scavenger tests were performed to determine the active species photogenerated by the ZnO nanoparticles involved in the degradation of RhB. Two scavengers were used: vitamin C and isopropyl alcohol (IPA) to quench the O_2_^–^ and ^•^OH activity, respectively. The tests were performed in the presence of the ZnO nanoparticles (NS, NF, and NU) annealed at 800 °C, exhibiting the best photocatalytic activity. Figure S8 illustrates the effect of Vitamin C and IPA on the photocatalytic degradation of RhB, compared with the photodegradation rate without scavengers. The obtained results indicate a decreased photodegradation rate for both used scavengers. However, the presence of Vitamin C results in a substantial decrease in the photodegradation rate, indicating that O_2_^–^ species have the most significant influence regardless of the ZnO morphology.

#### 3.4.2 ROS Species Generation

**Figure 9:**
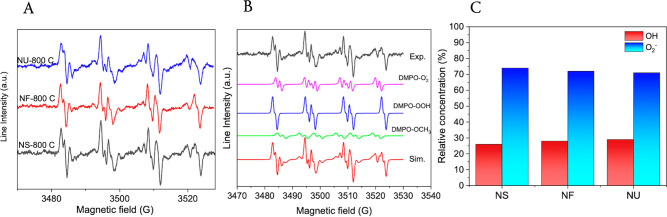
(A) Experimental spectra of DMPO spin adducts generated by all the samples; (B) Simulated spectra of DMPO spin adducts generated by NF-800; (C) Relative concentration of the spin adducts generated by the analyzed samples.

The photogeneration of reactive oxygen species by ZnO samples annealed at 800 °C was investigated by EPR spectroscopy coupled with the spin-trapping technique. The samples tested were those with the best photocatalytic activity under UV light irradiation. DMPO was used as a spin-trapping agent. The samples were dispersed in DMSO, illuminated for 25 min, and then measured by EPR. The spectra corresponding to the analyzed samples are shown in Figure 9. Since complex spectra were obtained, a simulation using a linear combination of different spin adducts was performed to highlight the reactive species generated. All spectra are composed of three main spin adducts, ^•^DMPO-OCH_3_ (a_N_ = 13.19 G, a_H_ = 7.9 G, a_H_ = 1.64 G), ^•^DMPO-OOH (a_N_ = 13.77 G, a_H_ = 11.7 G, a_H_ = 0.86 G) and, ^•^DMPO-O_2_^–^ (a_N_ = 12.78 G, a_H_ = 10.8 G, a_H_ = 1.33 G), but in different proportions. The spin adduct ^•^DMPO-CH_3_ appears because of the reaction between the ^•^OH radicals and the DMSO solvent, whereas the presence of ^•^DMPO-OOH is due to the interaction between DMPO and O_2_^–^ species. The simulations showed a relative concentration of O_2_^–^ superior (∼72%) to that corresponding to OH (∼28%) for all analyzed samples. These results follow the scavenger test results, which showed that O_2_^–^ species mainly achieve pollutant photodegradation.

**Figure 10:**
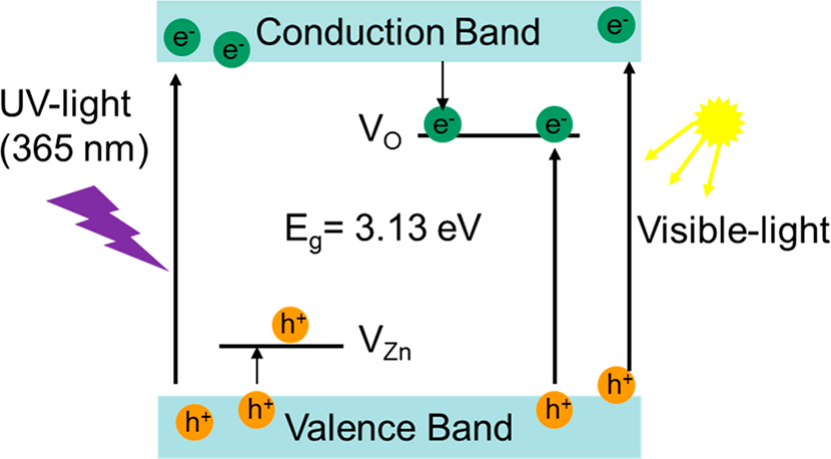
Schematic representation of the photocatalytic mechanism.

A photodegradation mechanism for RhB can be elaborated, considering the above-mentioned results. The schematic representation of the photocatalytic mechanism is presented in Figure 10, where UV light irradiation excites electrons from the valence band to the conduction band, forming electron–hole (e–h) pairs. If no recombination occurs, the holes from the valence band can interact with water molecules to form ^•^OH radicals. The conduction band electrons will create O_2_^–^ radicals by interacting with adsorbed oxygen. The presence of oxygen vacancies and other defects can inhibit the process of e–h pair recombination by capturing the photoinduced electrons and forming free or binding excitons, which can be observed as or conduct an increase in the PL signal intensity.^83^ In addition, the presence of oxygen vacancies facilitates the adsorption of O_2_ molecules from the solution, and electrons captured in oxygen vacancies will more easily interact with O_2_.^84^ Consequently, electrons trapped in defects/oxygen vacancies will also participate in the O_2_^–^ formation, consistent with the EPR spin-trapping results showing the generation of mainly O_2_^–^ radicals.

Besides, during visible irradiation, direct band-to-band excitation is much lower when compared to UV irradiation since visible light only comprises about 5% of UV radiation. So, under visible irradiation, most excitation transitions occur from the valence band to energy levels within the bandgap of ZnO caused by bulk and surface defects.

**Figure 11:**
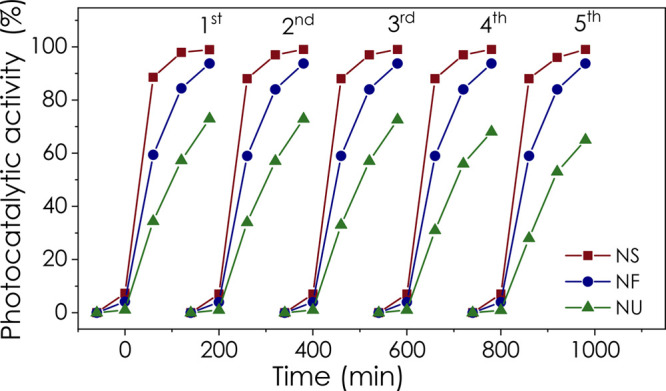
Reusability test of ZnO NS, NF, and NU annealed at 800 °C.

Finally, we have investigated the reusability of the ZnO nanopowder catalyst. As shown in Figure 11, the performance of ZnO NS and NF catalysts is very stable. The photocatalytic activity of ZnO NS and NF was almost identical after 5 cycles. On the other hand, the maximum photocatalytic activity of the NU catalyst decreased from 74 to 65% after 5 cycles. In light of all the data, we determined that ZnO NS nanocatalysts annealed at 800 °C under atmospheric conditions came to the forefront as a convenient and cost-effective catalyst for RhB degradation under UV light excitation. In future studies, the degradation performance can be improved by sensitizing this catalyst with small wavelength semiconductors or making heterojunctions. At the same time, the visible light sensitivity can be increased.

## 4 Conclusion

In this study, three different morphologies, nanoflower (NF), nano sponge (NS), and nano urchin-like (NU), ZnO nanostructures were synthesized successfully via a mild hydrothermal method. After synthesis, the samples were annealed in the atmosphere at 300, 600, and 800 °C. EDS analysis indicated that annealing at 300 °C resulted in Zn-rich samples for all morphologies. The Zn/O ratio decreased with increasing the annealing temperature. As evidenced by FTIR spectra, the intensities of C=O symmetric and asymmetric stretching of carboxylate ions increased with increasing the annealing temperature, indicating the complete transformation of crude products into ZnO. When the PL, EPR, BET, and XRD analysis results are examined, it is revealed that the defect structure, pore diameter, and crystallinity cumulatively affect the photocatalytic activity of ZnO nanocatalysts. As a result, to obtain high photocatalytic activity for rhodamine B degradation, it is necessary to develop a ZnO catalyst with fewer core defects, more oxygen vacancies, near band emission, and large crystallite size and pore diameter. ZnO NS-800 °C nanocatalyst had a 35.6 × 10^–3^ min^–1^ rate constant and excellent stability after a 5-cycle photocatalytic degradation of RhB.

## Supporting Information Available

Additional analysis, spectra, and crystal data (PDF)
