# Supplementary material for: Impact on the Photocatalytic Dye Degradation of Morphology and Annealing-Induced Defects in Zinc Oxide Nanostructures
Source: ACS Omega. 2023 Apr 17;8(17):14952–64. doi: 10.1021/acsomega.2c07412 (PMC10157689; doi:10.1021/acsomega.2c07412)
Supplement: Supplementary file 1 — ao2c07412_si_001.pdf [file ao2c07412_si_001.pdf]

Impact on the Photocatalytic Dye Degradation of Morphology and Annealing-Induced Defects in Zinc Oxide Nanostructures

Cigdem Tuc Altaf,[a] Tuluhan Olcayto Colak, [b] Arpad Mihai Rostas,[c]† Adriana Popa,[c] Dana Toloman,[c] Maria Suciu,[c] Nurdan Demirci Sankir,[a,b]§ Mehmet Sankir,[a,b]‡

[a] Department of Materials Science and Nanotechnology Engineering, TOBB University of Economics and Technology, SogutozuCaddesi No 43 Sogutozu 06560 Ankara, Turkey

[b] Micro and Nanotechnology Graduate Program, TOBB University of Economics and Technology, SogutozuCaddesi No 43 Sogutozu 06560 Ankara, Turkey

[c] National Institute for Research and Development of Isotopic and Molecular Technologies-INCDTIM, 67-103 Donat, 400293 Cluj-Napoca, Romania

Corresponding authors: †arpad.rostas@infim.ro ; §nsankir@etu.edu.tr ; ‡ msankir@etu.edu.tr

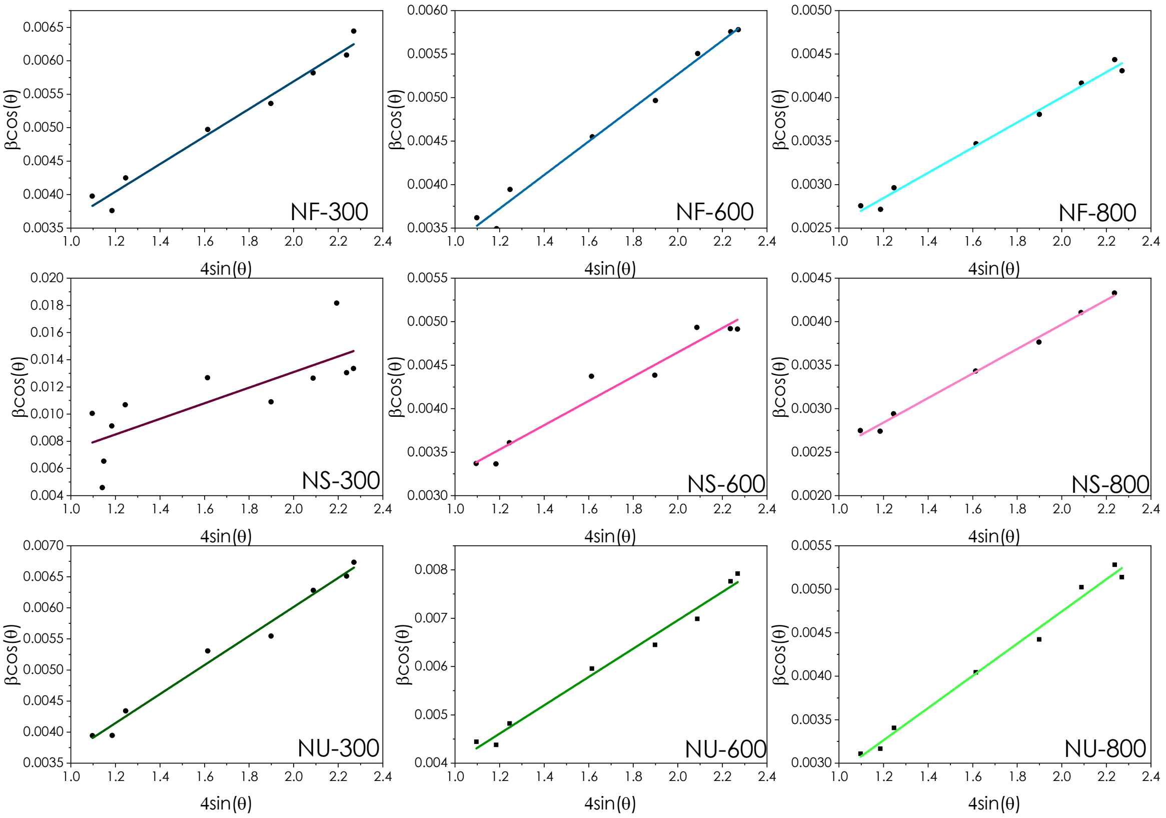

Figure S1. Williamson-Hall analysis of ZnO having different morphologies and annealing temperatures**.**

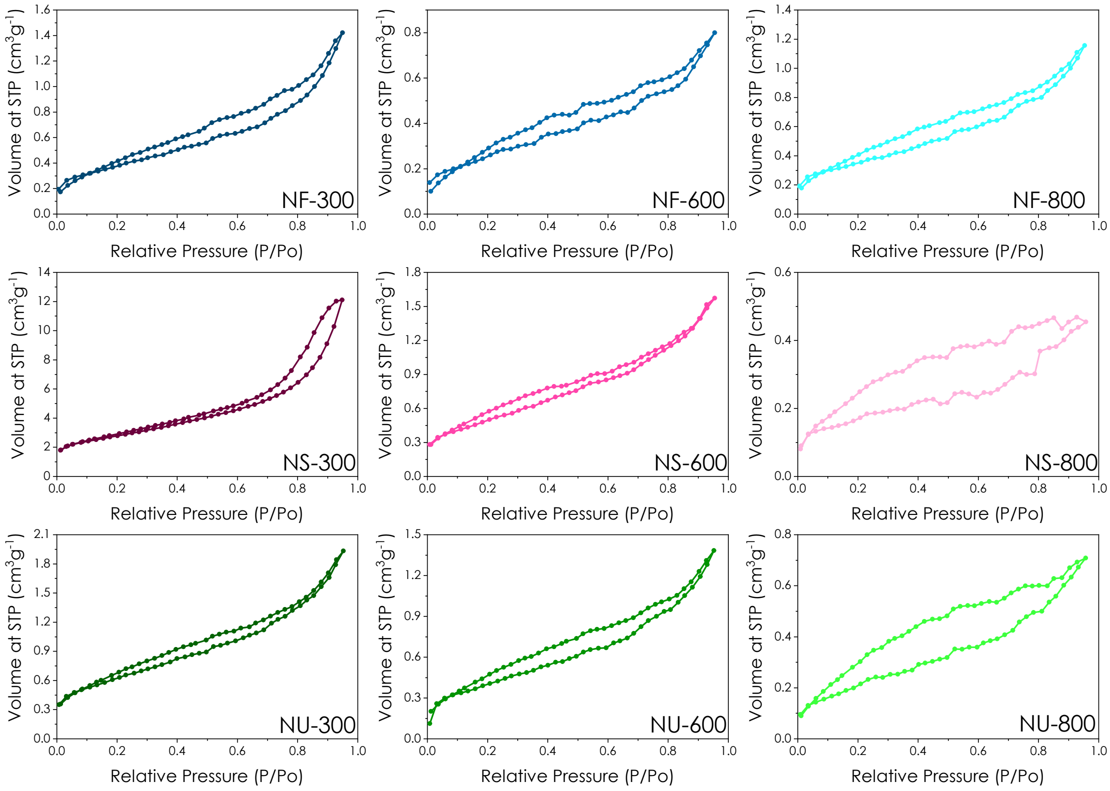

Figure S2. N2 sorption data of samples at their given temperatures.

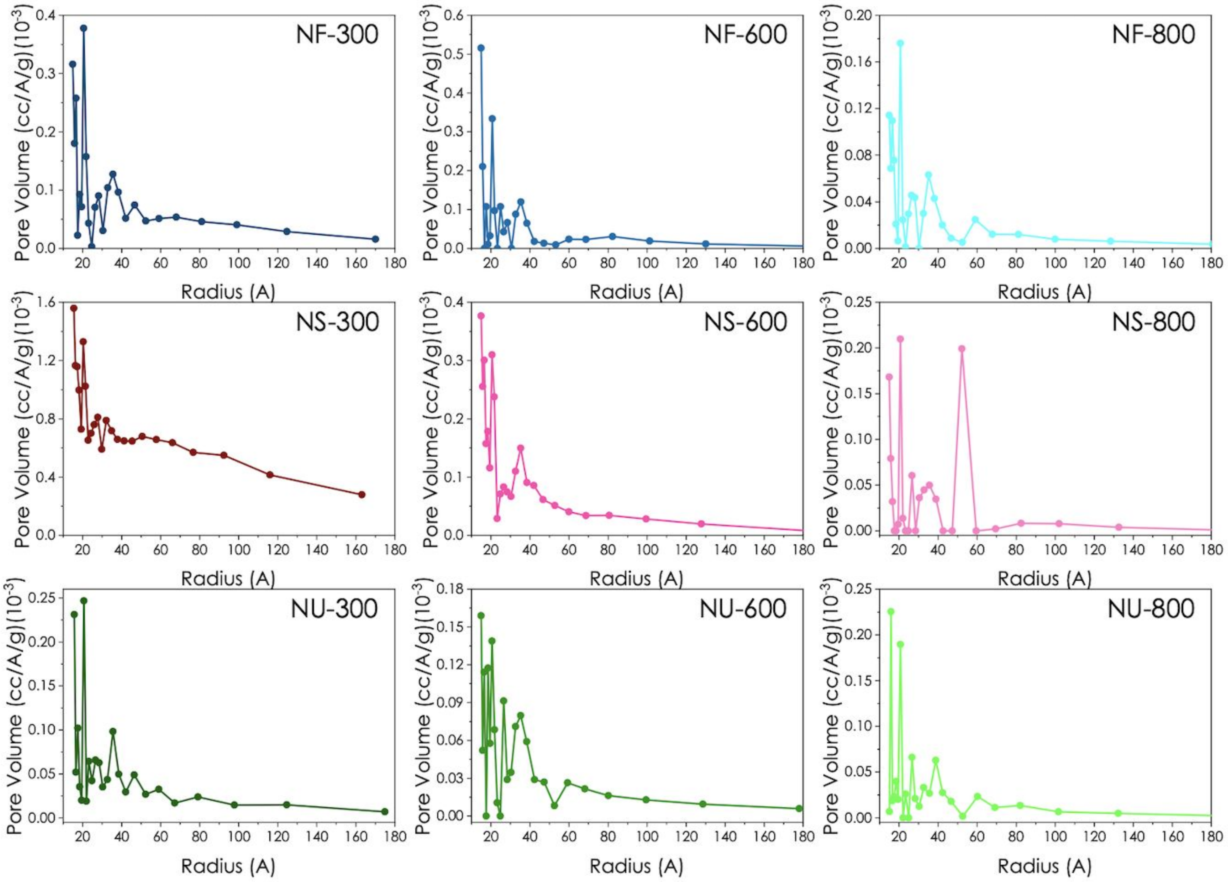

Figure S3. BJH pore size distribution plots of samples at their given temperatures.

Table S1. The summary of crystal properties and particle size of ZnO nanoparticles.

Sample	FWHM (°)	Grain size (nm)	Strain, ε	Surface area(m^2g^{-1})	Pore radius (Å)
NF-300	0.32987	29.52479	0.00206	5.26	20.57
NF-600	0.30517	31.95247	0.00193	3.67	14.96
NF-800	0.23217	41.92914	0.00145	1.93	20.69
NS-300	0.60086	16.71509	0.00574	51.69	15.48
NS-600	0.27399	35.06138	0.0014	6.04	14.92
NS-800	0.21355	45.49008	0.00252	2.06	20.73
NU-300	0.34589	28.36589	0.00234	3.81	20.65
NU-600	0.32596	33.00901	0.00293	2.99	14.93
NU-800	0.27271	35.9645	0.00185	1.58	15.80

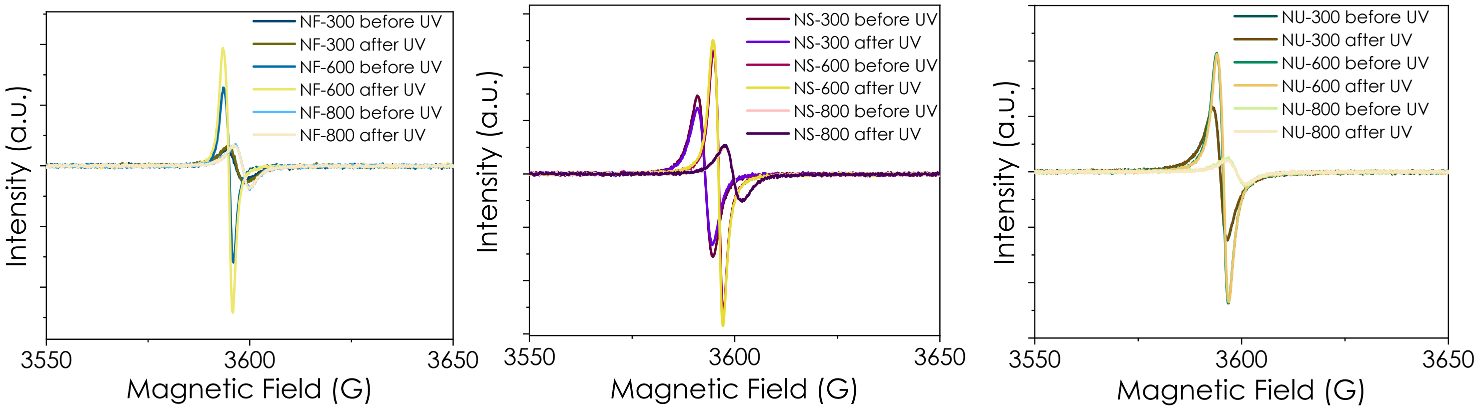

Figure S4. EPR spectra of the samples before and after UV radiation

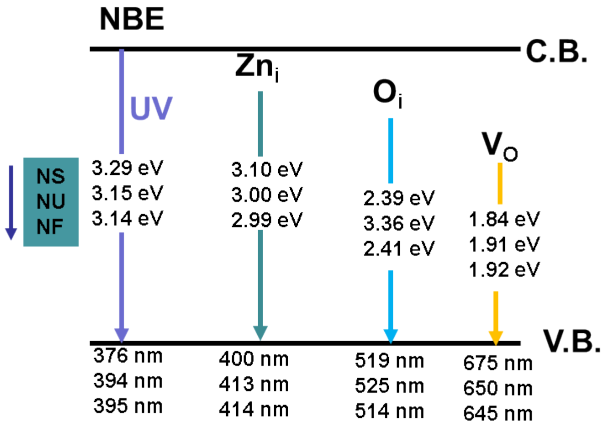

Figure S5. Energy level diagram showing observed defect levels in ZnO nanoparticles

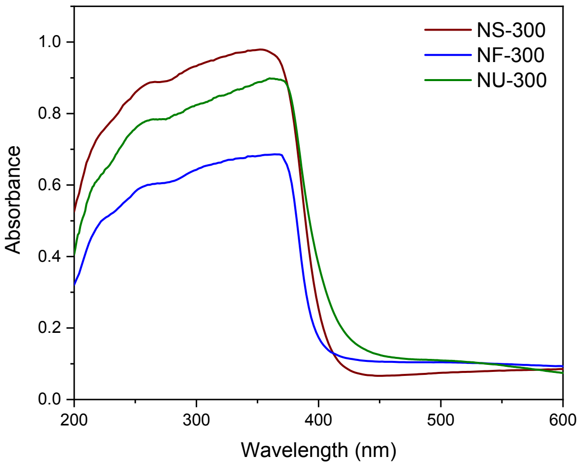

Figure S6. UV-Vis absorbance spectra of NS-300, NF-300, and NU-300 samples.

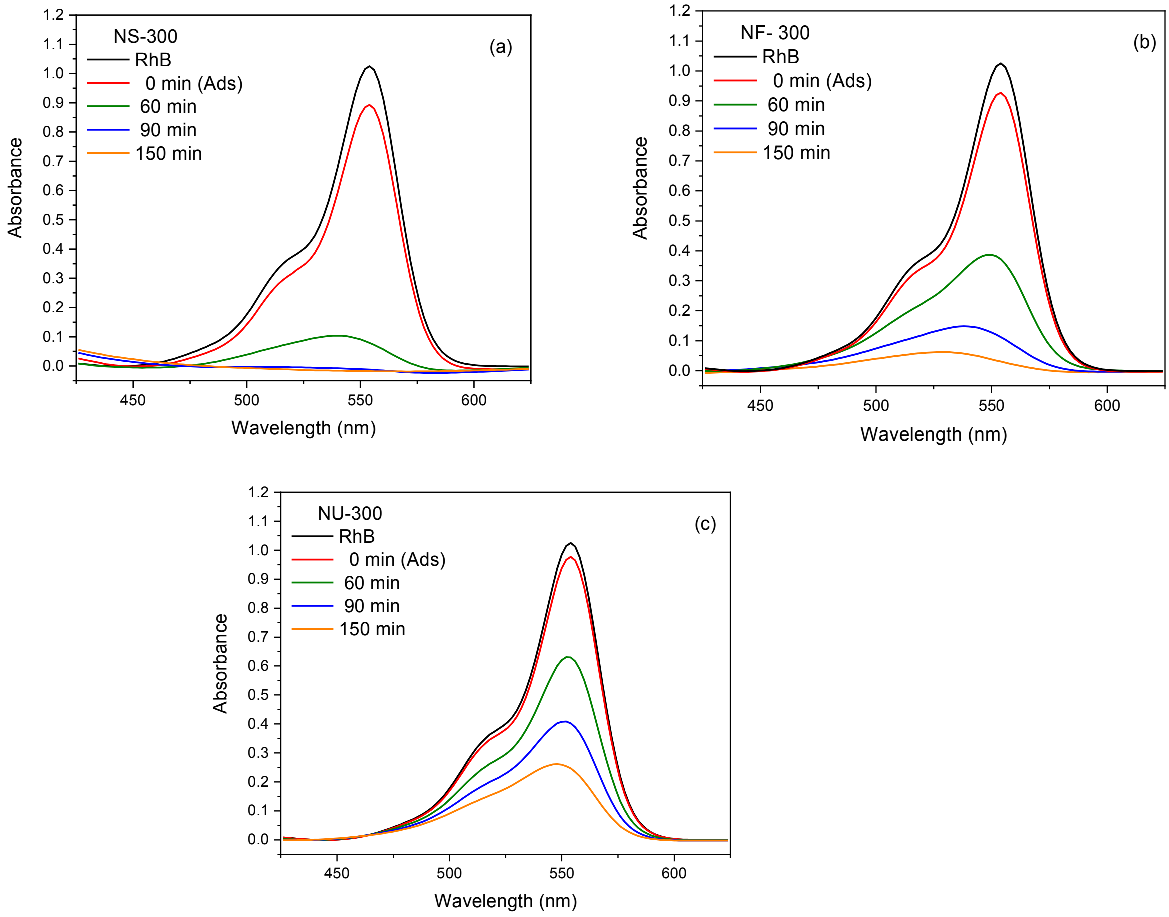

Figure S7. UV–vis absorption spectra of RhB aqueous solution with (a) NS-300, (b) NF-300, (c) NU-300 sample at different irradiation time intervals.

Table S2. Photocatalytic RhB degradation performance for all samples.

**Sample**	**k x 10^{-3}**	**R^2**
NS-300	25.28	0.9577
NS-600	31.2	0.9537
NS-800	35.6	0.964
NF-300	15.24	0.9996
NF-600	11.19	0.9925
NF-800	24.95	0.9523
NU-300	7.19	0.9992
NU-600	6.22	0.9461
NU-800	12.44	0.9998

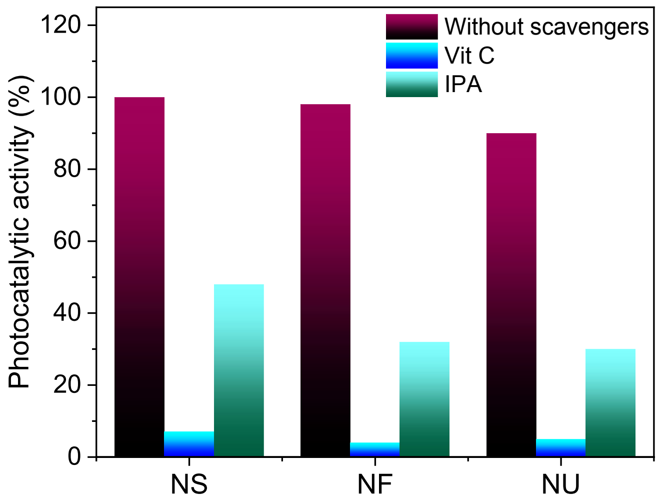

Figure S8. The effect of scavengers on the RhB degradation process.
